# Trajectory Tracking Control for Flexible-Joint Robot Based on Extended Kalman Filter and PD Control

**DOI:** 10.3389/fnbot.2019.00025

**Published:** 2019-05-24

**Authors:** Tianyu Ma, Zhibin Song, Zhongxia Xiang, Jian S. Dai

**Affiliations:** ^1^Key Laboratory of Mechanism Theory and Equipment Design of Ministry of Education, Tianjin University, Tianjin, China; ^2^School of Mechanical Engineering, Tianjin University, Tianjin, China; ^3^Centre for Robotics Research School of Natural and Mathematical Sciences, King's College London, London, United Kingdom

**Keywords:** flexible joint, extended Kalman filter, closed-loop PD controller, lyapunov stability, trajectory tracking

## Abstract

The robot arm with flexible joint has good environmental adaptability and human robot interaction ability. However, the controller for such robot mostly relies on data acquisition of multiple sensors, which is greatly disturbed by external factors, resulting in a decrease in control precision. Aiming at the control problem of the robot arm with flexible joint under the condition of incomplete state feedback, this paper proposes a control method based on closed-loop PD (Proportional-Derivative) controller and EKF (Extended Kalman Filter) state observer. Firstly, the state equation of the control system is established according to the non-linear dynamic model of the robot system. Then, a state prediction observer based on EKF is designed. The state of the motor is used to estimate the output state, and this method reduces the number of sensors and external interference. The Lyapunov method is used to analyze the stability of the system. Finally, the proposed control algorithm is applied to the trajectory control of the flexible robot according to the stability conditions, and compared with the PD control algorithm based on sensor data acquisition under the same experimental conditions, and the PD controller based on sensor data acquisition under the same test conditions. The experimental data of comparison experiments show that the proposed control algorithm is effective and has excellent trajectory tracking performance.

## Introduction

With the increasing use of robots in the fields of industry, the rehabilitation, aviation, and marine exploration, the demand for robots that can adapt to complex environments and enable human-robot interaction is increasing, which introduces flexible structure onto robotic joints (Schiavi et al., 2008; Grioli et al., 2015). The general flexible design is to adopt elastic elements and harmonic reducers to reduce the rigidity of robot joint (Zhu and Schutter, 1999). For higher usage requirements, the flexible cushioning is realized by a variable stiffness driver, and the stiffness performance can be adjusted in a wide range (Wolf and Hirzinger, 2008; Ham et al., 2009; Jafari et al., 2011; Torreaiba and Udelman, 2016). The reduction in the stiffness of the robot increases the safety, but at the same time leads to a reduction in the dynamic performance of the structure, which includes slow response, delayed control, and limited bandwidth. It makes the flexible robot based on variable stiffness more difficult to control (Hogan, 1985; Hurst et al., 2004; Erler et al., 2014).

In recent years, research on the control of flexible-joint robots has become more and more attractive. A spring-damping mode was first proposed to simplify the flexible-joint, and at the same time, the flexible-joint is divided into two subsystems for control by integral flow and perturbation theory (Spong, 1987), and the sliding mode controller was designed (Sira-ramirez and Spong, 1988). Then, based on this model, the robustness analysis of the feedback linearization method suitable for this simplified model was given (Grimm, 1990). A method for compensation system with parameter uncertainty was desidned (Zeman et al., 1997; Ge et al., 1998). To reduce the number of differentials required for motion equations and task equations the design method for task space tracking control and proposed an implicit numerical integration method was proposed which is effective (Ider and Ozgoren, 2000). The impedance model formed on the basis of the simplified spring- damping model is considered to be a typical compliant control strategy, that is, cross-compatibility is achieved through the interaction between the robot system and the external environment, allowing a certain degree of partial movement of the actual trajectory and a given trajectory (Jamwal et al., 2016; Losey et al., 2016). In order to control the robot trajectory more precisely, the effects of flexible properties on the dynamic performance of robots was systematically analyzed (Zaher and Megahed, 2015), and the control problems of flexible-joint robot position, torque, and impedance control based on passivity were studied (Albu- schäffer et al., 2007). However, these control models are somewhat stretched when it comes to external disturbances and non-linear systems.

In order to solve the problem of jitter and friction in flexible robot tracking control, the adaptive CFBC (command-filtered backstepping control) was proposed to improve tracking accuracy (Pan et al., 2018). For the purpose of solving the interference problem of control, the ADRC (Active Disturbance Rejection Control) was designed based on the modern control theory which relies on the accurate mathematical model, that can effectively control the system with uncertainty and external interference (Han, 2009). However, more parameters need to be calculated for non-linear systems. The disturbance observer proposed by Ohnishi in 1987 can be used for the disturbance that is difficult to measure in the system (Nakao et al., 1987). The external disturbance is estimated by the input amount and the feedback value of the inner loop, as the observation compensation amount, and it is added to the control to cancel the actual interference (Sariyildiz and Ohnishi, 2013; Sariyildiz et al., 2015). However, as the order for the filter increases, the large phase lag causes the system to be underdamped and even makes the system unstable.

Throughout the above control methods, the classic PD controller, with its “natural” anti-interference and model-independence, is widely used in the control of series elastic actuators by matching feedforward control (Zhu et al., 2012). It is worth noting that the PD controller relies on the data feedback of the system, therefore, its performance can be greatly affected by external disturbance introduced via sensory collection, and moreover, the use of multiple sensors increases the cost and structural design difficulty of the robot.

In order to solve the above problems, this paper first proposes the state estimation of non-linear stiffness-driven flexible robot with EKF, as it has good convergence and low computational complexity, and can handle system uncertainty and external disturbances in real time (Reif et al., 1998; Lightcap and Banks, 2010). This method can reduce the use of the sensor and introduce noise covariance into the observer design to reduce estimation error. Considering the external disturbance, a closed-loop PD controller based on EKF is designed to achieve precise position control that requires only joint motor side position and speed measurements.

This paper is organized as follows. The model, principle and dynamics analysis of the flexible-joint robot are introduced in section Robotic Prototype and its Dynamic Model. Subsequently, the design of the closed-loop PD controller and EKF state observer is introduced in section EKF Based Controller. The stability analysis based on Lyapunov method is presented in section Lyapunov Stability Analysis. The experimental results are presented in section Experimental Results. Finally, a conclusion is provided in section Conclusion.

## Robotic Prototype and Dynamic Model of Robot

### Robotic Prototype

**Figure 1:**
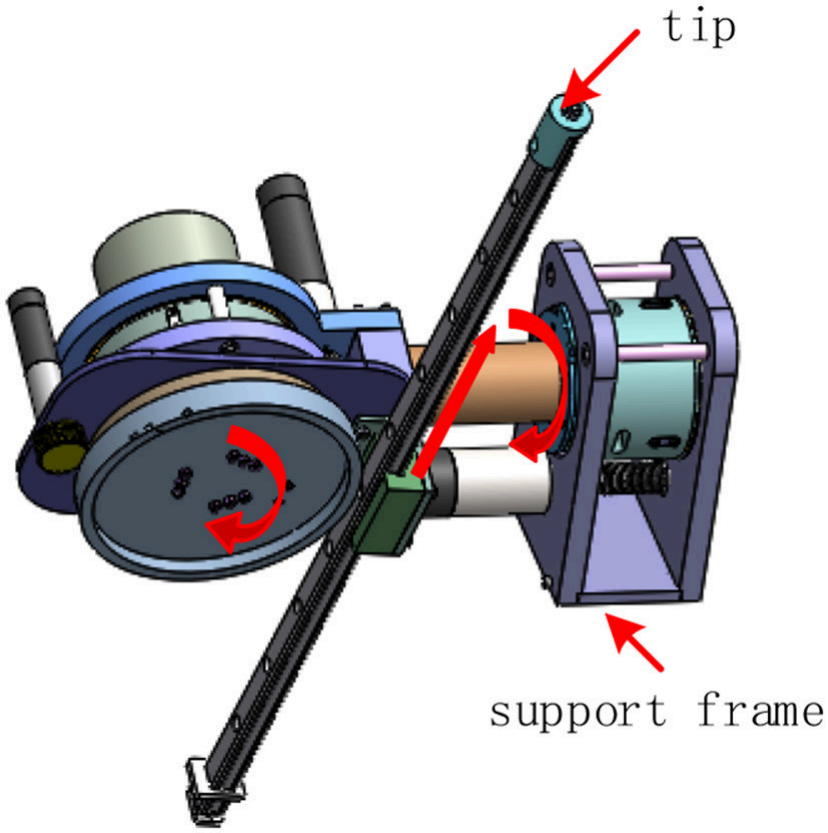
Robotic prototype.

In order to demonstrate our method, a 3-DOF robot with flexible joint is introduced in this section to verify the algorithm. As shown in Figure 1, the robot can be regarded as an open-chain series connected by two rotations and one moving joint. In the linkage system, each joint is driven by a non-linear actuator, and the third joint turns the rotation of the joint into translation through the slider and the guide rail.

Since the control target of this paper is the trajectory output of the tip, based on the configuration of the robot, the variables of the three joints are respectively calculated by the inverse kinematics according to the desired trajectory output, and the three joints are respectively controlled according to the timing.

**Figure 2:**
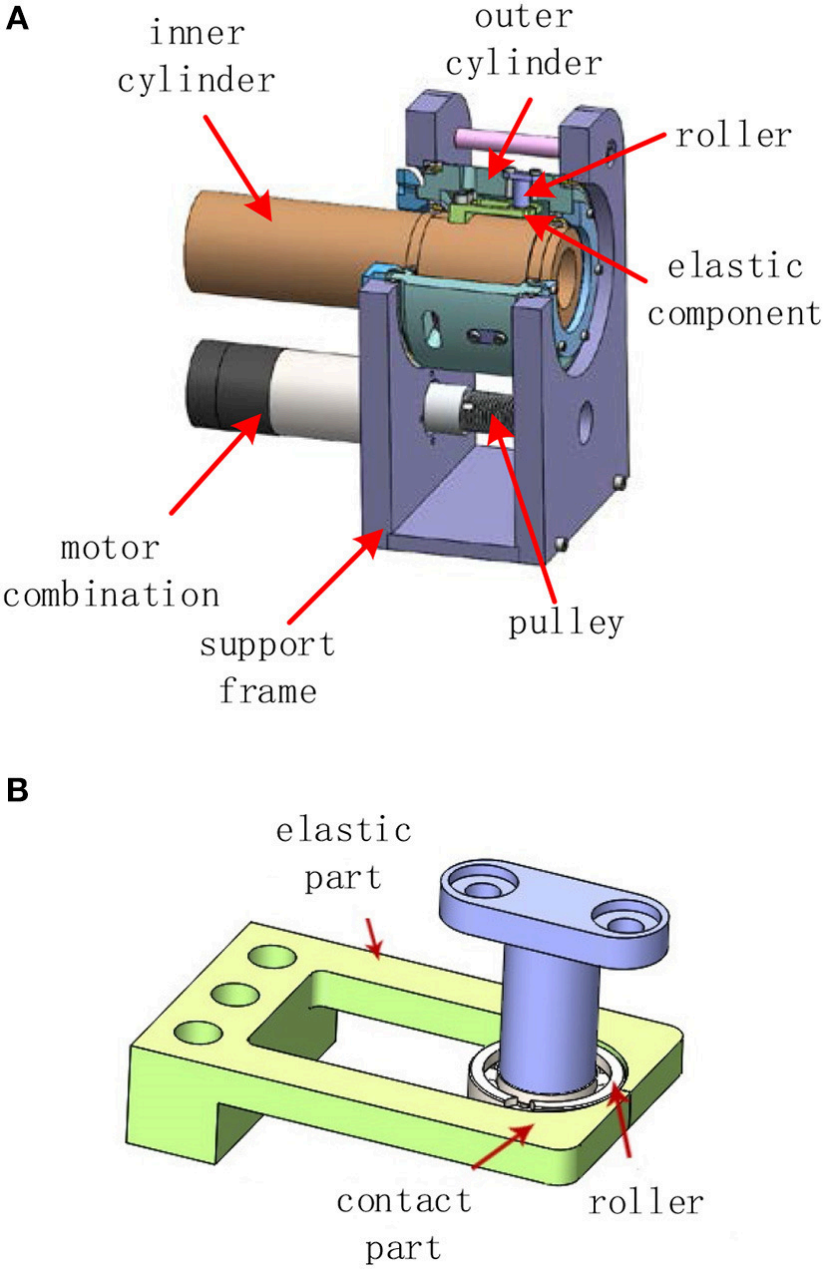
Actuator structure **(A)** Main structure. **(B)** Elastic structure.

The structure of the three actuators is basically the same. In this paper, the first joint actuator is taken as an example to introduce the structure. As shown in Figure 2A, the structure of the non-linear stiffness actuator mainly includes the support frame, motor combination (DC brushless motor, reducer, encoder), pulley, outer cylinder, wire, inner cylinder, and elastic structure. The motor is the power source driving the pulley, which drives the outer cylinder through the wire. The inner wall of the outer cylinder is provided with a radially uniform roller shaft, and the rotation of the roller presses and fixes the bidirectional elastic structure fixed on the inner cylinder, thereby driving the inner cylinder to rotate.

The non-linear elastic structure is shown in Figure 2B, the non-linear elastic structure is the core mechanical structure of the non-linear stiffness actuator and consists of a roller and an elastic component. The elastic component is composed of symmetrical elastic units, each of which consists of a cantilever beam portion and a contact portion. Although the two parts are made of the same material, when the roller presses the contact portion, the deformation of the contact portion can be regarded as a rigid structure, and the cantilever beam portion is deformed, and the deflection and the deflection angle of the elastic portion will cause the position of the contact point to change. There is a certain mapping relationship between the positional change of the contact point caused by the deflection perpendicular to the end of the cantilever beam portion and the pressing force.

In addition, the change of the deflection angle also affects the vertical component of the contact point position. Therefore, the relationship between the positional change in the vertical direction of the contact point and the pressing force is no longer a simple proportional relationship, but the combination of the deflection and the deflection angle. In short, the contour curve of the contact surface determines the relationship between the pressing force and displacement of the contact point, that is, the stiffness variation curve. The specific design scheme and the non-linear mechanism have been deeply studied by the researcher group (Lan and Song, 2016), and this paper will not go into details.

### Dynamic Model

Non-linear stiffness actuator can be divided into power systems, transmission systems, elastic structures, and external loads. The power system is the motor combination, which mainly includes the motor rotor and the gear reducer. The equivalent moment of inertia of the motor combination can be obtained from the dynamics model of the motor combination. The dynamic equation of the rotor of the motor is:

(1) $$\begin{array}{l} J_{r}{\ddot{\theta }}_{r}+b_{r}{\dot{\theta }}_{r}=\tau_{m}-\tau_{r} \end{array}$$

where *J*_*r*_ and *b*_*r*_ are the moment of inertia and damping of the rotor of the motor respectively; ${\dot{\theta }}_{r}$ and ${\ddot{\theta }}_{r}$ are the angular velocity and angular acceleration of the rotor of the motor respectively; τ_*m*_ is the torque generated by the rotor of the motor; τ_*r*_ is the torque output by the rotor of the motor.

The dynamic equation of the motor reducer is:

(2) $$\begin{array}{l} J_{g}{\ddot{\theta }}_{g}+b_{g}{\dot{\theta }}_{g}=R_{1}\tau_{r}-\tau_{g} \end{array}$$

where *J*_*g*_ and *b*_*g*_ are the moment of inertia and damping of the motor reducer respectively; ${\dot{\theta }}_{g}$ and ${\ddot{\theta }}_{g}$ are the angular velocity and angular acceleration of the motor reducer respectively; *R*_1_ is the reduction ratio; τ_*g*_ is the torque output by the motor reducer.

Since the motor rotor and the gear reducer are rigidly connected, the following relationship is used:

(3) $$\begin{array}{l} \frac{{\ddot{\theta }}_{r}}{{\ddot{\theta }}_{g}}=\frac{{\dot{\theta }}_{r}}{{\dot{\theta }}_{g}}=\frac{\theta_{r}}{\theta_{g}}=R_{1} \end{array}$$

where θ_*r*_ and θ_*g*_ are the motor rotor angle and the gear reducer angle respectively. Combined with Equations (1–4) can be obtained:

(4) $$\begin{array}{l} \left(J_{r}+\frac{J_{g}}{R_{1}^{2}}\right){\ddot{\theta }}_{r}+\left(b_{r}+\frac{b_{g}}{R_{1}^{2}}\right){\dot{\theta }}_{r}=\tau_{m}-\frac{\tau_{g}}{R_{1}} \end{array}$$

**Figure 3:**
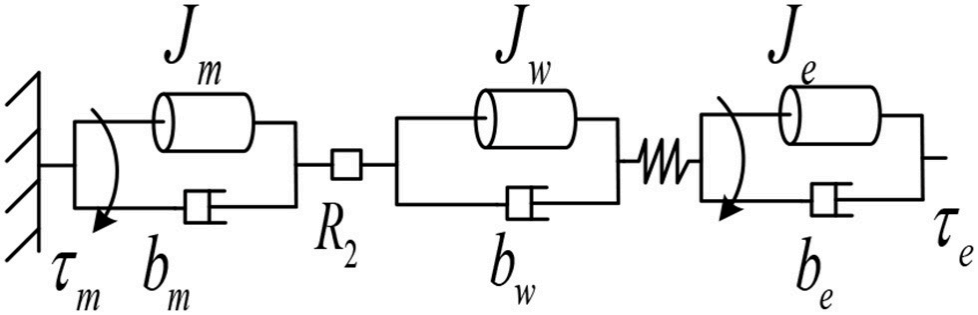
Schematic diagram of the actuator.

The schematic diagram of the actuator from the motor combination to the output is shown in Figure 3.

The dynamic equation of the outer cylinder section is:

(5) $$\begin{array}{l} J_{w}{\ddot{\theta }}_{w}+b_{w}{\dot{\theta }}_{w}=R_{2}\tau_{g}-\tau_{k} \end{array}$$

where: *J*_*w*_ and *b*_*w*_ are the moment of inertia and damping of the outer drum, respectively; ${\ddot{\theta }}_{w}$ and ${\dot{\theta }}_{w}$ are the angular velocity and angular acceleration of the output shaft of the outer drum of the non-linear stiffness drive, respectively; *R*_2_ is the reduction ratio of the wire drive, and the relationship between the angular velocity and the angular velocity of the outer cylinder is:

(6) $$\begin{array}{l} \frac{{\ddot{\theta }}_{g}}{{\ddot{\theta }}_{w}}=\frac{{\dot{\theta }}_{g}}{{\dot{\theta }}_{w}}=\frac{\theta_{g}}{\theta_{w}}=R_{2} \end{array}$$

where θ_*w*_ is the angle of rotation of the outer cylinder for the non-linear stiffness drive, simultaneous Equations (4–6) can obtain:

(7) $$\begin{array}{l} \left(J_{r}+\frac{1}{{R_{1}}^{2}}J_{g}+\frac{J_{w}}{{R_{1}}^{2}{R_{2}}^{2}}\right){\ddot{\theta }}_{r}+\left(b_{r}+\frac{1}{{R_{1}}^{2}}b_{g}+\frac{b_{w}}{{R_{1}}^{2}{R_{2}}^{2}}\right){\dot{\theta }}_{r}\\ \;=\tau_{m}-\frac{\tau_{k}}{R_{1}R_{2}} \end{array}$$

Then the equivalent dynamic equation of the motor assembly to the elastic part is:

(8) $$\begin{array}{l} J_{eq}{\ddot{\theta }}_{r}+b_{eq}{\dot{\theta }}_{r}=\tau_{m}-\frac{\tau_{k}}{R_{1}R_{2}} \end{array}$$

where $J_{eq}=J_{r}+\frac{1}{{R_{1}}^{2}}J_{g}+\frac{J_{w}}{{R_{1}}^{2}{R_{2}}^{2}}$ is the actuator equivalent inertia and $b_{eq}=b_{r}+\frac{1}{{R_{1}}^{2}}b_{g}+\frac{b_{w}}{{R_{1}}^{2}{R_{2}}^{2}}$ is the actuator equivalent damping.

The dynamic equation of the outer cylinder part is:

(9) $$\begin{array}{l} J_{e}{\ddot{\theta }}_{e}+b_{e}{\dot{\theta }}_{e}=\tau_{k}-\tau_{e} \end{array}$$

where: *J*_*e*_ and *b*_*e*_ are the moment of inertia and damping of the external load, respectively; τ_*e*_ is the output torque of the drive; ${\ddot{\theta }}_{e}$ and ${\dot{\theta }}_{e}$ are the angular velocity and angular acceleration of the external load, respectively.

## Ekf Based Controller

### Equation of State

It can be seen from the above formula that the dynamic equation from the motor to the output shaft without considering the external torque input is:

(10) $$\begin{array}{l} J_{e}{\ddot{\theta }}_{e}+b_{e}{\dot{\theta }}_{e}=R_{1}R_{2}\left(\tau_{m}-J_{eq}{\ddot{\theta }}_{r}-b_{eq}{\dot{\theta }}_{r}\right) \end{array}$$

**Figure 4:**
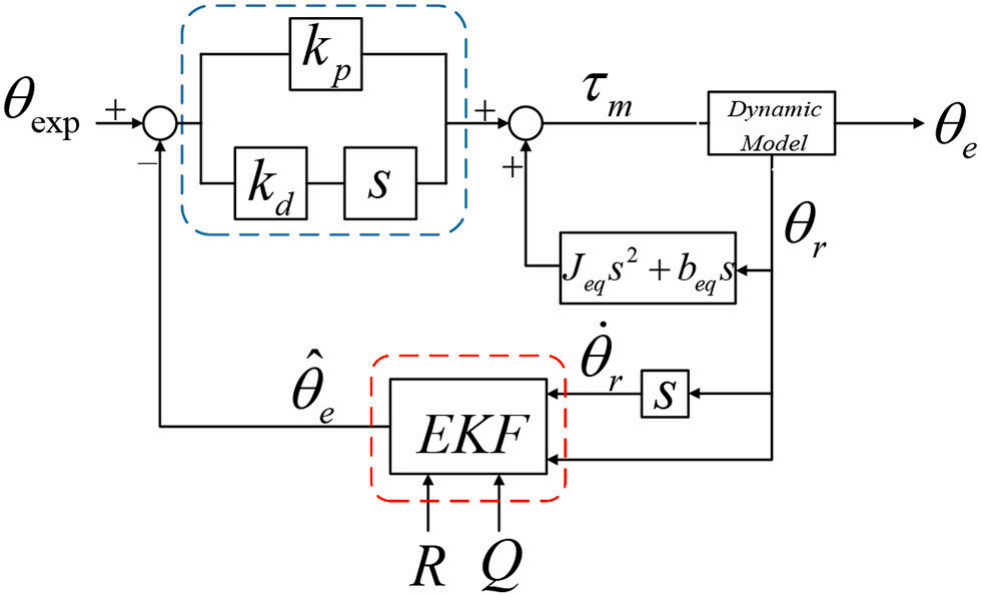
EKF based PD controller.

The EKF based PD controller is shown in Figure 4.

In the control system, τ_*m*_ can be considered to consist of two parts:

(11) $$\begin{array}{l} \tau_{m}=\tau_{dy}+\tau_{d} \end{array}$$

where τ_*dy*_ is the part consumed by the equilibrium dynamics, and its expression is:

(12) $$\begin{array}{l} \tau_{dy}-J_{eq}{\ddot{\theta }}_{r}-b_{eq}{\dot{\theta }}_{r}=0 \end{array}$$

τ_*d*_ is the torque required for the end output, which is output by the PD controller. The design expression is as follows:

(13) $$\begin{array}{l} \tau_{d}=K_{p}\left(\theta_{e}-\theta_{\exp}\right)+K_{d}\left({\dot{\theta }}_{e}-{\dot{\theta }}_{\exp}\right) \end{array}$$

where *K*_*p*_ is the proportional stiffness coefficient and *K*_*d*_ is the differential damping coefficient, substituting (11–13) into (10), we can get:

(14) $$\begin{array}{l} J_{e}{\ddot{\theta }}_{e}+b_{e}{\dot{\theta }}_{e}=R_{1}R_{2}[J_{eq}{\ddot{\theta }}_{r}+b_{eq}{\dot{\theta }}_{r}+K_{p}(\theta_{e}-\theta_{\exp})+\\ \;K_{d}({\dot{\theta }}_{e}-{\dot{\theta }}_{\exp})-J_{eq}{\ddot{\theta }}_{r}-b_{eq}{\dot{\theta }}_{r}] \end{array}$$

It can be simplified to:

(15) $$\begin{array}{l} {\ddot{\theta }}_{e}+a_{1}{\dot{\theta }}_{e}+a_{2}\theta_{e}=b_{0}+b_{1}{\dot{\theta }}_{\exp}+b_{2}\theta_{\exp} \end{array}$$

where $a_{1}=\frac{b_{e}-R_{1}R_{2}K_{d}}{J_{e}}$; $a_{2}=\frac{R_{1}R_{2}K_{p}}{-J_{e}}$; *b*_0_ = 0; $b_{1}=\frac{R_{1}R_{2}K_{d}}{-J_{e}}$; $b_{2}=\frac{R_{1}R_{2}K_{p}}{-J_{e}}$.

The formula is a typical input-output equation with a derivative term, so the state variables are chosen as follows:

(16) $$\begin{array}{l} \theta_{1}=\theta_{e}-b_{0}\theta_{\exp}\\ \theta_{2}={\dot{\theta }}_{1}-h_{1}\theta_{\exp} \end{array}$$

where *h*_1_ = *b*_1_ − *a*_1_*b*_0_, then the equation of state of the system is:

(17) $$\begin{array}{l} {\dot{\theta }}_{1}=\theta_{2}+h_{1}\theta_{\exp}\\ {\dot{\theta }}_{2}=-a_{2}\theta_{1}-a_{1}\theta_{2}+h_{2}\theta_{\exp} \end{array}$$

where *h*_2_ = (*b*_2_ − *a*_2_*b*_0_) − *a*_1_*h*_1_, rewritten into a matrix form:

(18) $$\begin{array}{l} \left[\begin{array}{c} {\dot{\theta }}_{1}\\ {\dot{\theta }}_{2} \end{array}\right]=\left[\begin{array}{cc} 0 & 1\\ -a_{2} & -a_{1} \end{array}\right]\left[\begin{array}{c} \theta_{1}\\ \theta_{2} \end{array}\right]+\left[\begin{array}{c} h_{1}\\ h_{2} \end{array}\right]\theta_{\exp} \end{array}$$

### Ekf State Observer Design

According to the control frame we designed in the previous section, we can see that the PD position controller based on EKF requires the output shaft angle and angular velocity to be the feedback amount. In order to solve the sensor's measurement interference, cost, and structural design issues, we use the EKF state observer to predict the angle of the output shaft and the angular velocity. The inputs are only the angle and angular velocity of the motor. The angular acceleration is obtained from the first derivative of the angular velocity and filtered by a low-pass filter to eliminate high-frequency interference.

According to [29], the relationship between the output torque of the elastic component and the rotor angle of the motor and the output angle of the shutdown section is as follows:

(19) $$\begin{array}{l} \tau_{k}=0.15{\left(\frac{\theta_{r}}{R_{1}R_{2}}-\theta_{e}\right)}^{5}-0.23{\left(\frac{\theta_{r}}{R_{1}R_{2}}-\theta_{e}\right)}^{4}+\\ 1.78{\left(\frac{\theta_{r}}{R_{1}R_{2}}-\theta_{e}\right)}^{3}+0.67\left(\frac{\theta_{r}}{R_{1}R_{2}}-\theta_{e}\right) \end{array}$$

Then the overall dynamic equation can be written as:

(20) $$\begin{array}{l} R_{1}R_{2}\left(\tau_{m}-J_{eq}{\ddot{\theta }}_{r}+b_{eq}{\dot{\theta }}_{r}\right)=0.15{\left(\frac{\theta_{r}}{R_{1}R_{2}}-\theta_{e}\right)}^{5}-\\ 0.23{\left(\frac{\theta_{r}}{R_{1}R_{2}}-\theta_{e}\right)}^{4}+\;1.78{\left(\frac{\theta_{r}}{R_{1}R_{2}}-\theta_{e}\right)}^{3}+0.67\left(\frac{\theta_{r}}{R_{1}R_{2}}-\theta_{e}\right) \end{array}$$

(21) $$\begin{array}{l} J_{e}{\ddot{\theta }}_{e}+b_{e}{\dot{\theta }}_{e}=0.15{\left(\frac{\theta_{r}}{R_{1}R_{2}}-\theta_{e}\right)}^{5}-0.23{\left(\frac{\theta_{r}}{R_{1}R_{2}}-\theta_{e}\right)}^{4}+\\ 1.78{\left(\frac{\theta_{r}}{R_{1}R_{2}}-\theta_{e}\right)}^{3}+0.67\left(\frac{\theta_{r}}{R_{1}R_{2}}-\theta_{e}\right) \end{array}$$

According to the formula, it can be seen that the experimental platform of this paper is a typical non-linear system. According to the EKF observation method in document [27], combined with the control objectives of this paper, the state variables are defined as:

(22) $$\begin{array}{l} x={\left[\begin{array}{cccc} x_{1} & x_{2} & x_{3} & x_{4} \end{array}\right]}^{T}={\left[\begin{array}{cccc} \theta_{e} & {\dot{\theta }}_{e} & \theta_{r} & {\dot{\theta }}_{r} \end{array}\right]}^{T} \end{array}$$

Deriving it to time *t* and substituting it into the dynamics equation, we get the state function *f*(*x*):

(23) $$\begin{array}{l} f\left(x\right)=\frac{\partial x}{\partial t}=\left[\begin{array}{c} {\dot{\theta }}_{e}\\ M\\ {\dot{\theta }}_{r}\\ N \end{array}\right] \end{array}$$

where $M=\frac{1}{J_{e}}\left(\tau_{k}-b_{e}{\dot{\theta }}_{e}\right)$; $N=\frac{1}{J_{eq}}\left(\tau_{m}-b_{eq}{\dot{\theta }}_{r}-\frac{\tau_{k}}{R_{1}R_{2}}\right)$ We can obtain state function by partial differentiation of equation (23):

(24) $$\begin{array}{l} F\left(t\right)=\frac{\partial f\left(x\right)}{\partial x}=\left[\begin{array}{cccc} 0 & 1 & 0 & 0\\ F_{1} & F_{2} & F_{3} & 0\\ 0 & 0 & 0 & 1\\ F_{4} & 0 & F_{5} & F_{6} \end{array}\right] \end{array}$$

where $F_{1}=\frac{1}{J_{e}}▪\frac{\partial \tau_{k}}{\partial \theta_{e}}$; $F_{2}=-\frac{b_{e}}{J_{e}}$; $F_{3}=\frac{1}{J_{e}}▪\frac{\partial \tau_{k}}{\partial \theta_{r}}$; $F_{4}=-\frac{1}{J_{eq}R_{1}R_{2}}▪\frac{\partial \tau_{k}}{\partial \theta_{e}}$; $F_{5}=-\frac{1}{J_{eq}R_{1}R_{2}}▪\frac{\partial \tau_{k}}{\partial \theta_{r}}$; $F_{6}=-\frac{b_{eq}}{J_{eq}}$

Define the observation vector as:

(25) $$\begin{array}{l} h\left(x\right)=\left[\begin{array}{c} x_{3}\\ x_{4} \end{array}\right]=\left[\begin{array}{c} \theta_{r}\\ {\dot{\theta }}_{r} \end{array}\right] \end{array}$$

Then the state observation matrix is:

(26) $$\begin{array}{l} H\left(t\right)=\frac{\partial h\left(x\right)}{\partial x}=\left[\begin{array}{cccc} 0 & 0 & 1 & 0\\ 0 & 0 & 0 & 1 \end{array}\right] \end{array}$$

The EKF iteration formula is as follows:

(27) $$\begin{array}{l} \dot{\hat{x}}=f\left(\hat{x},\tau_{m}\right)+G\left(t\right)\left(h\left(x\right)-h\left(\hat{x}\right)\right) \end{array}$$

(28) $$\begin{array}{l} \dot{P}\left(t\right)=F\left(t\right)P\left(t\right)+P\left(t\right)F^{T}\left(t\right)+Q\left(t\right)-\\ \;P\left(t\right)H^{T}\left(t\right)R^{-1}\left(t\right)H\left(t\right)P\left(t\right) \end{array}$$

(29) $$\begin{array}{l} G\left(t\right)=P\left(t\right)H^{T}\left(t\right)R^{-1}\left(t\right) \end{array}$$

where $\hat{x}$ is the predicted estimate of ***x***; ***Q***(*t*) and ***R***(*t*) are the process noise and measurement noise obeying the Gaussian distribution; ***G***(*t*) is the extended Kalman gain, and ***P***(*t*) is the predicted error covariance. Without external disturbance, we can accurately capture the information we need to know through the sensor, but in the presence of external noise and unknown factors, our prediction will be biased. After each prediction, the EKF state observer adds new uncertainty to establish a connection with external disturbances, that is, the measurement covariance ***R***(*t*) and system covariance ***Q***(*t*) obeying the Gaussian distribution. The end of the robot may be affected by other disturbances. By establishing different observation matrices, we can reasonably estimate and compensate for the disturbance force experienced by the robot joints.

## Lyapunov Stability Analysis

According to the research of extended Kalman filter, the stability of the control system of the flexible joint robot proposed in this paper is that the overall PD control system is stable, and the EKF observer is stable. The system stability analysis in this paper is divided into the following two steps:

Step 1: Proof of PD controller stability.

The Lyapunov method is used to prove the stability of the controller. Therefore, the previous system state Equation (18) can be written as:

(30) $$\begin{array}{l} \dot{\theta }=A\theta +B\theta_{\exp} \end{array}$$

where $\theta \text{=}{\left[\begin{array}{cc} \theta_{\text{1}} & \theta_{\text{2}} \end{array}\right]}^{T};A\text{=}\left[\begin{array}{cc} 0 & 1\\ -a_{2} & -a_{1} \end{array}\right];B\text{=}\left[\begin{array}{c} h_{1}\\ h_{2} \end{array}\right]$

Define the Lyapunov equation as:

(31) $$\begin{array}{lll} V\left(\theta \right) & \text{=} & \theta^{T}\text{U}\theta \end{array}$$

(32) $$\begin{array}{l} A^{\tau }\text{U}+\text{U}A=-E \end{array}$$

where ***E*** is unit matrix, then *U* can be contained:

(33) $$\begin{array}{l} U=\left[\begin{array}{cc} \frac{a_{1}^{2}+a_{2}^{2}+a_{2}}{2a_{1}a_{2}} & \frac{1}{2a_{2}}\\ \frac{1}{2a_{2}} & \frac{1+a_{2}}{2a_{1}a_{2}} \end{array}\right] \end{array}$$

It is a positive definite matrix. Then the derivative of the Lyapunov equation is:

(34) $$\begin{array}{l} \dot{V}\left(\theta \right)={\dot{\theta }}^{T}U\theta +\theta^{T}U\dot{\theta }=\frac{a_{1}^{2}+a_{2}^{2}+a_{2}}{a_{1}a_{2}}{\dot{\theta }}_{1}\theta_{1}\\ \;+\frac{1}{a_{2}}\left({\dot{\theta }}_{2}\theta_{1}+{\dot{\theta }}_{1}\theta_{2}\right)+\frac{1+a_{2}}{a_{1}a_{2}}{\dot{\theta }}_{2}\theta_{2} \end{array}$$

Through the adjustment of the PD parameters, it can make $\dot{V}\left(\theta \right)<0$. The PD controller system is stable, and the intermediate calculation process will not be described in detail herein.

Step 2: Proof of EKF observer stability

Defining observation error: $\mu =x-\hat{x}$.

Expand ***f***(***x***) and ***h***(***x***):

(35) $$\begin{array}{l} f\left(x\right)-f\left(\hat{x}\right)=\text{F}\left(t\right)\mu +\alpha \end{array}$$

(36) $$\begin{array}{l} h\left(x\right)-h\left(\hat{x}\right)=H\left(t\right)\mu +\beta \end{array}$$

where ***α*** and ***β*** are the higher order terms of ***μ***, then:

(37) $$\begin{array}{l} \dot{\mu }=\left[F\left(t\right)-G\left(t\right)H\left(t\right)\right]\mu +\alpha -G\left(t\right)\beta \end{array}$$

Define the Lyapunov equation as:

(38) $$\begin{array}{l} W=\mu^{T}\Pi \mu \end{array}$$

where Π = **P**^−1^, then:

(39) $$\begin{array}{l} \dot{W}=\mu^{\text{T}}\dot{\Pi }\mu +\mu^{T}\left(\text{F}-\text{GH}\right)\Pi \mu +\\ \mu^{T}\Pi \left(\text{F}-\text{GH}\right)\mu +2\mu^{T}\Pi \left(\alpha -\text{G}\beta \right) \end{array}$$

Assumption: $\|\alpha \|\leq k_{\alpha }{\left\|\mu \right\|}^{2}$, $\|\beta \|\leq k_{\beta }{\left\|\mu \right\|}^{2}$,$\|\text{H}\|\leq \bar{h}$, $\underset{-}{p}E\leq P\leq \bar{p}E$,$\underset{-}{q}E\leq \text{Q}$,$\underset{-}{r}E\leq R$, where *k*_α_, *k*_β_, $\bar{h}$, $\underset{-}{p}$, $\bar{p}$, $\underset{-}{q}$ and $\underset{-}{r}$ are positive constants.

Lemma: According to the assumptions, there are ε > 0 and κ > 0, then:

(40) $$\begin{array}{l} \mu^{\tau }\Pi \alpha -\mu^{\tau }\Pi \text{G}\beta \leq \kappa {\left\|\mu \right\|}^{3} \end{array}$$

for any ‖**μ**‖ that satisfies ‖**μ**‖ ≤ ε

Proof: According to **Π** = ***P***^−1^ and assumption, it can be obtained that:

(41) $$\begin{array}{l} \frac{1}{\bar{p}}{\left\|\mu \right\|}^{2}\leq W\leq \frac{1}{\underset{-}{p}}{\left\|\mu \right\|}^{2} \end{array}$$

Using triangular inequalities, ***G*** = ***PH***^*T*^**R**^−1^, Π = ***P***^*−1*^, and ‖**μ**‖ ≤ ε, it can be obtained that:

(42) $$\begin{array}{l} \left\|\mu^{\tau }\Pi \alpha -\mu^{\tau }\Pi G\beta \|\leq \|\mu^{\tau }\Pi \alpha \|+\|\mu^{\tau }{\text{H}}^{\tau }R^{-1}\beta \right\| \end{array}$$

Inequality can be obtained as follows according to assumption:

(43) $$\begin{array}{lll} \|\mu^{\tau }\Pi \alpha -\mu^{\tau }\Pi \text{G}\beta \|\leq \|\mu \|\frac{k_{\alpha }}{\underset{-}{p}}{\left\|\mu \right\|}^{2} & + & \|\mu \|\frac{\bar{h}k_{\beta }}{\underset{-}{r}}{\left\|\mu \right\|}^{2} \end{array}$$

(44) $$\begin{array}{l} \kappa =\frac{k_{\alpha }}{\underset{-}{p}}+\frac{\bar{h}k_{\beta }}{\underset{-}{r}} \end{array}$$

According to the lemma and ***G*** = ***PH**^τ^**R*****^−1^**, we can obtain that:

(45) $$\begin{array}{l} \dot{W}\leq -2iW+\left(-\frac{\underset{-}{q}}{{\bar{p}}^{2}}+2\kappa \|\mu \|\right){\left\|\mu \right\|}^{2} \end{array}$$

where *i* > 0, for any ‖*μ*‖ that satisfies $\|\mu \|\leq \varepsilon^{\prime }=\min\left(\varepsilon ,\frac{\underset{-}{q}}{4\kappa {\bar{p}}^{2}}\right)$, we can obtain that:

(46) $$\begin{array}{l} \dot{W}\left(t\right)\leq -\frac{\underset{-}{q}}{2{\bar{p}}^{2}}{\left\|\mu \left(t\right)\right\|}^{2}-2iW\left(t\right)=\left(-2i-\frac{\underset{-}{q}\underset{-}{p}}{2{\bar{p}}^{2}}\right)W\left(t\right) \end{array}$$

By using separation variable method, we can obtain that

(47) $$\begin{array}{l} W\left(t\right)\leq W\left(0\right)e^{(-2i(-2i-\frac{\underset{-}{q}\underset{-}{p}}{2{\bar{p}}^{2}})t} \end{array}$$

so Ẇ(*t*) < 0. According to the inequality $\frac{1}{\bar{p}}{\left\|\mu \right\|}^{2}\leq W\leq \frac{1}{\underset{-}{p}}{\left\|\mu \right\|}^{2}$, we can get the solution:

(48) $$\begin{array}{l} \|\mu \left(t\right)\|\leq \sqrt{\frac{\bar{p}}{\underset{-}{p}}}\|\mu \left(0\right)\|e^{-\left(i+\frac{\underset{-}{q}\underset{-}{p}}{4{\bar{p}}^{2}}\right)t} \end{array}$$

That is, the EKF state observer is exponentially stable.

In summary, step 1 and step 2 respectively prove that the PD control system is stable and the EKF state observer is stable. Therefore, the stability of the incomplete state feedback control system of the entire flexible joint robot is proved.

## Experimental Results

### Experimental Setup

In this part, the proposed control algorithm is applied to the prototype to prove the feasibility and stability of the control algorithm to compare with the sensor-based PD trajectory controller under the same experimental conditions.

**Figure 5:**
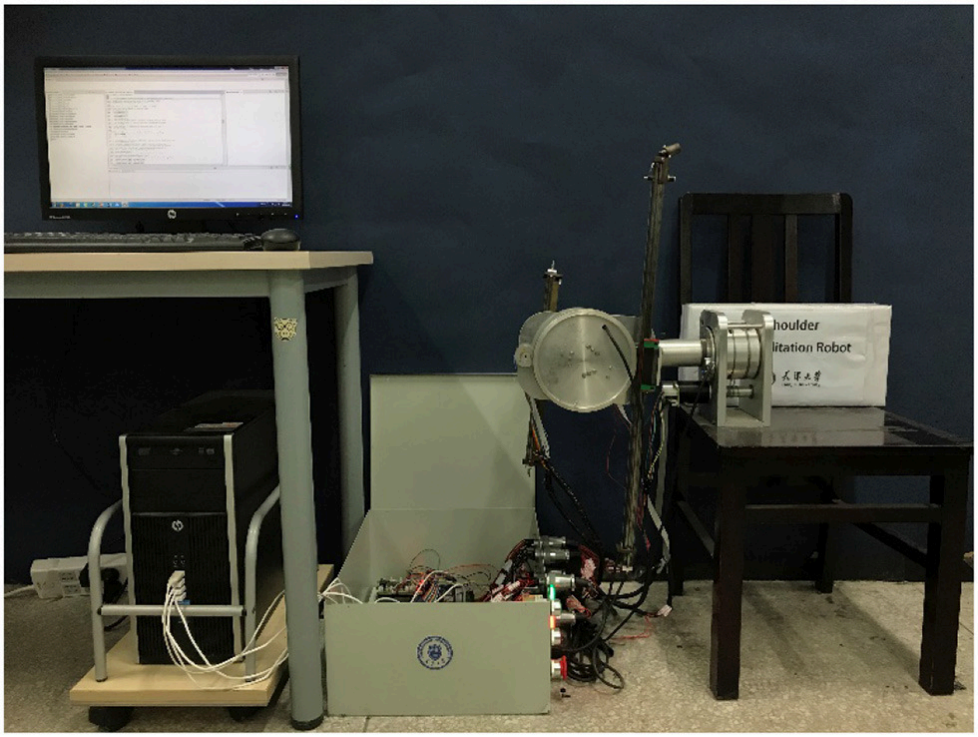
The entire experimental platform.

A 64-bit-Windows-8.1-based host computer with an Intel Core i7 processor @2.40 GHz and 8-GB RAM is used to run the Kalman estimation and calculate the input torque to the motor. The control algorithm is able to operate on an execution rate of 1 kHz using Visual C++ 2010, which is enough for real-time applications. The DSP board is used to read, process, and calculate the signal from the motor encoder and transmit it to the computer, that is, obtain the real-time position information of the motor through the QEP module of the DSP chip. Then the results calculated by the host computer are sent to the DSP board through a RS232 serial port. The DSP board then converts the input torque command into a PWM wave signal to drive the motor, and the A-D electromagnetic tracking system (trakSTAR, produced by NDI) is used to measure the position of the robot end. Through the USB cable, the location data is sent to the host for comparative verification of the experimental results. The entire experimental platform is shown in Figure 5.

### Experimental Data

**Figure 6:**
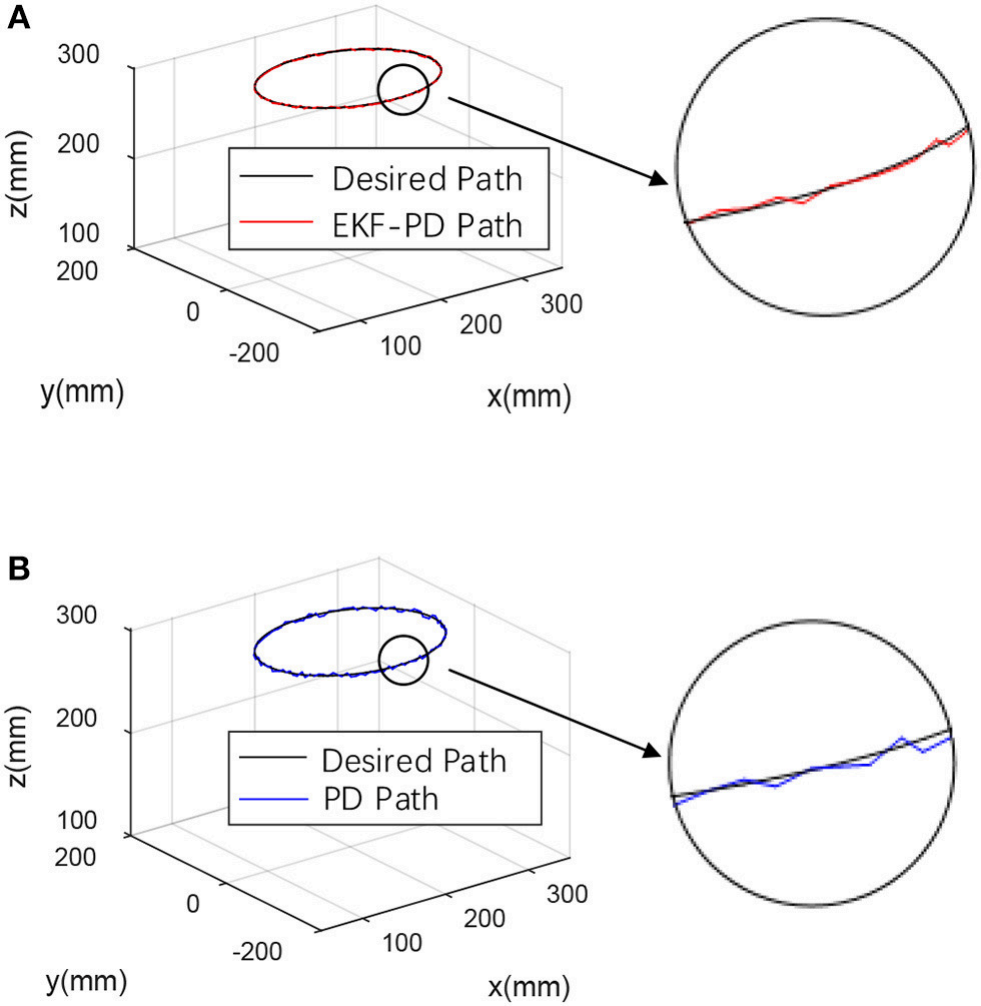
Experimental results **(A)** EKF-based PD controller experimental result. **(B)** Sensor-based PD controller experimental result.

For the fairness of the experiment and the validity of the comparison verification, the experiment was carried out in the same environment using the same machine, and the same PD controller parameters were used. The desired trajectory is a closed circular trajectory. The trajectory tracking results are shown in Figure 6.

To further analyze the experimental data, define the error mean square error:

(49) $$\begin{array}{l} RMSE=\sqrt{\left(\overset{T}{\underset{t=0}{\sum }}{\left\|\xi_{e}\left(t\right)-\xi_{\exp}\left(t\right)\right\|}_{2}\right)/T} \end{array}$$

where ξ_*e*_(*t*) and ξ_exp_(*t*) represent the actual trajectory and the desired trajectory, respectively.

**Figure 7:**
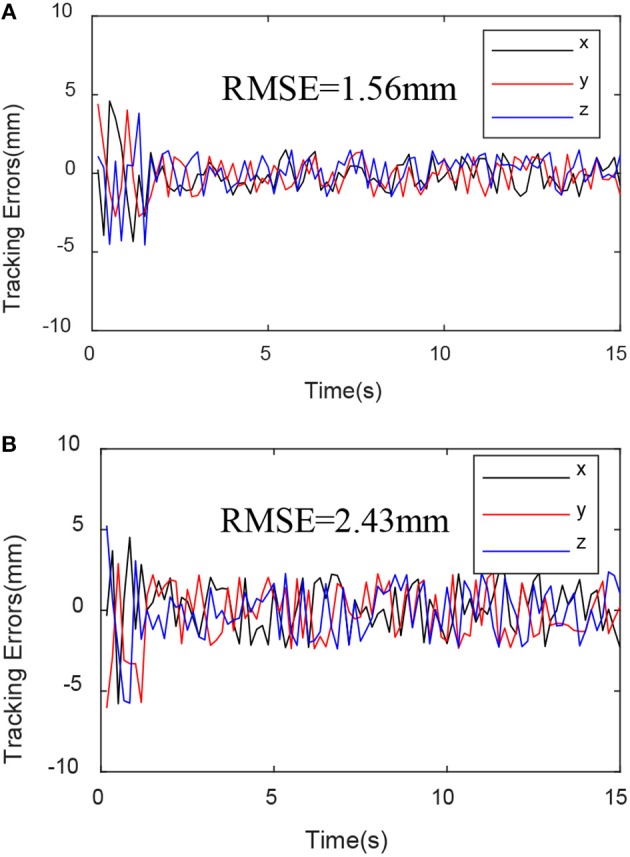
Trajectory tracking variance mean square. **(A)** EKF-based PD controller trajectory tracking variance mean square. **(B)** Sensor-based PD controller trajectory tracking variance mean square.

**Figure 8:**
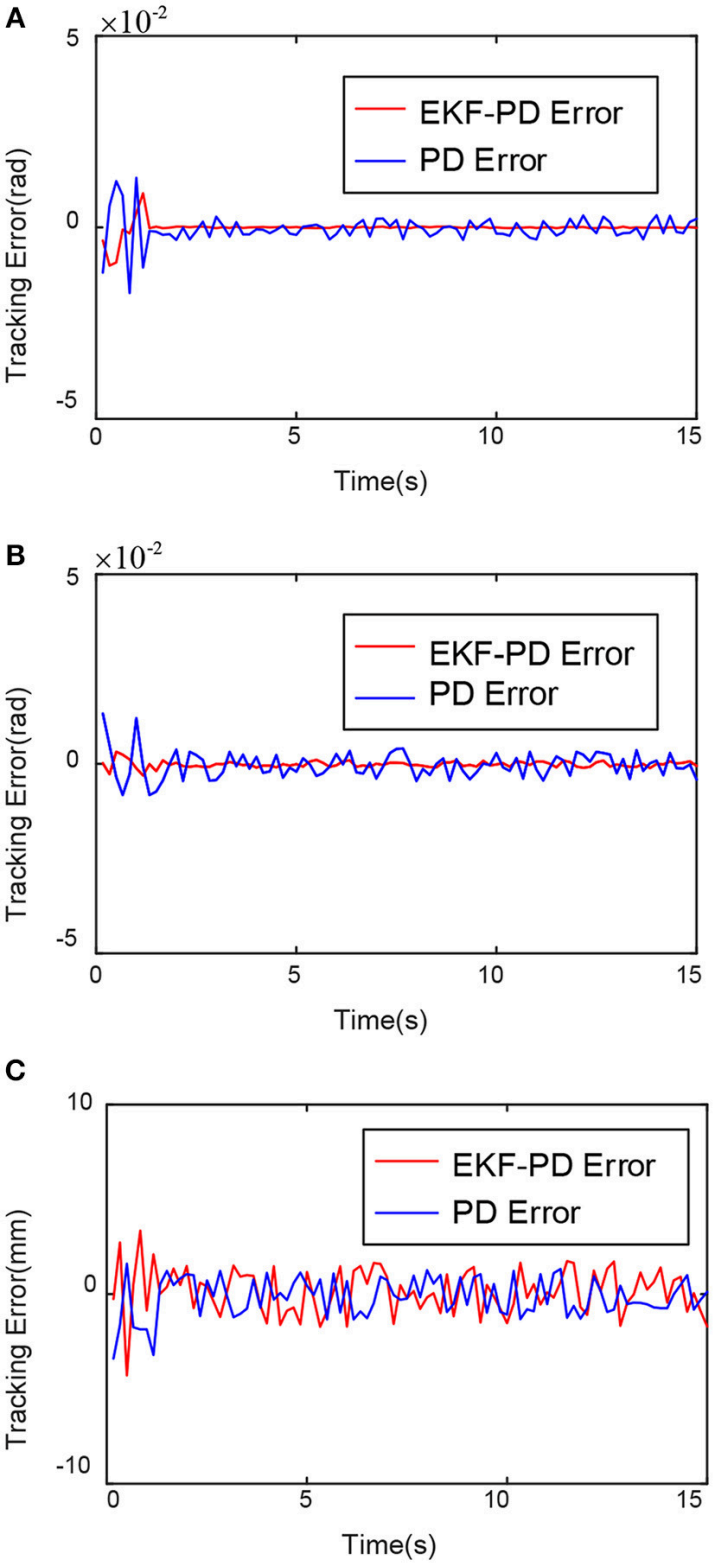
Comparison of tracking error of each joint. **(A)** First joint tracking error comparison. **(B)** Second joint tracking error comparison. **(C)** Third joint tracking error comparison.

Through the experimental results shown in Figures 6–8, we can see that the mean square value of the trajectory tracking error of the PD controller based on EKF is smaller, and tracking error of each joint is also smaller, which indicates that under the same conditions, the control algorithm proposed in this paper has better control effect.

**Figure 9:**
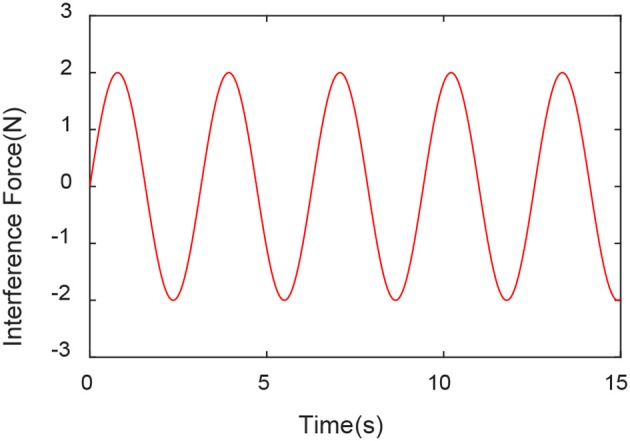
Disturbance Force.

**Figure 10:**
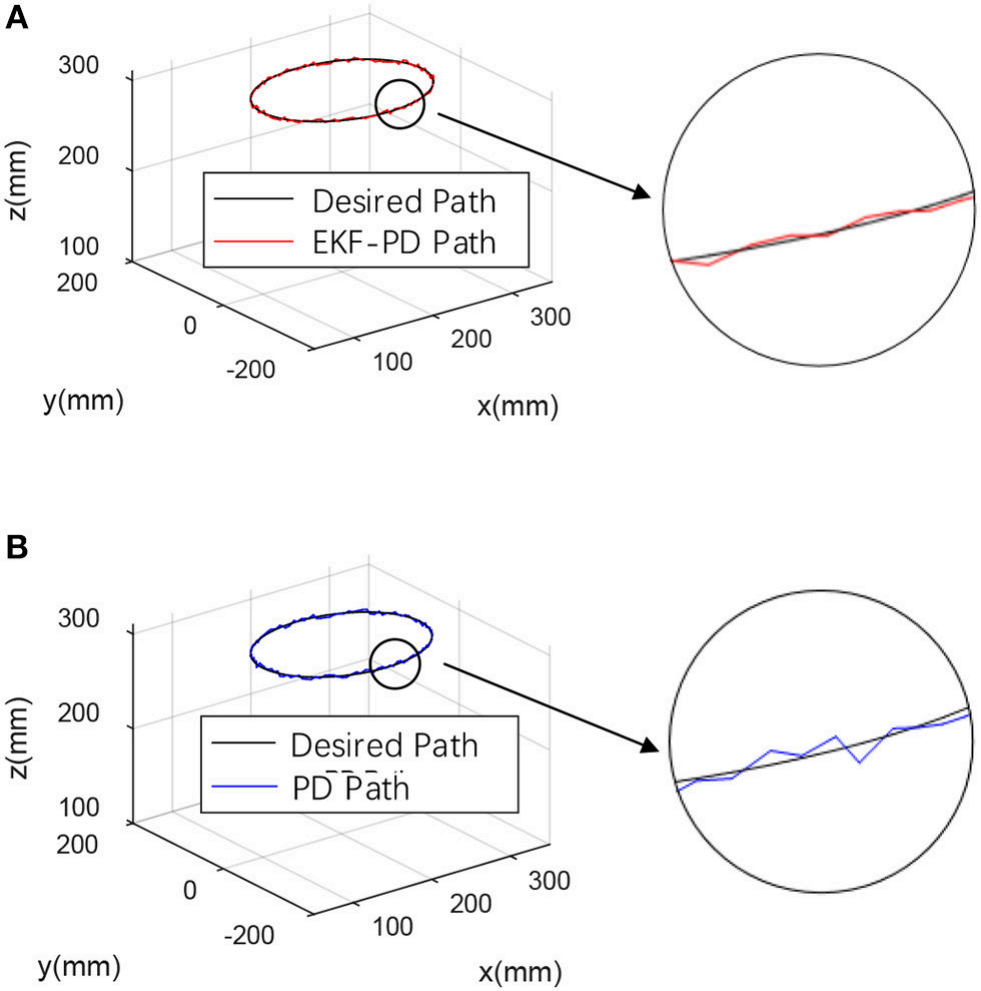
Experimental results with disturbance force **(A)** EKF-based PD controller experimental result. **(B)** Sensor-based PD controller experimental result.

**Figure 11:**
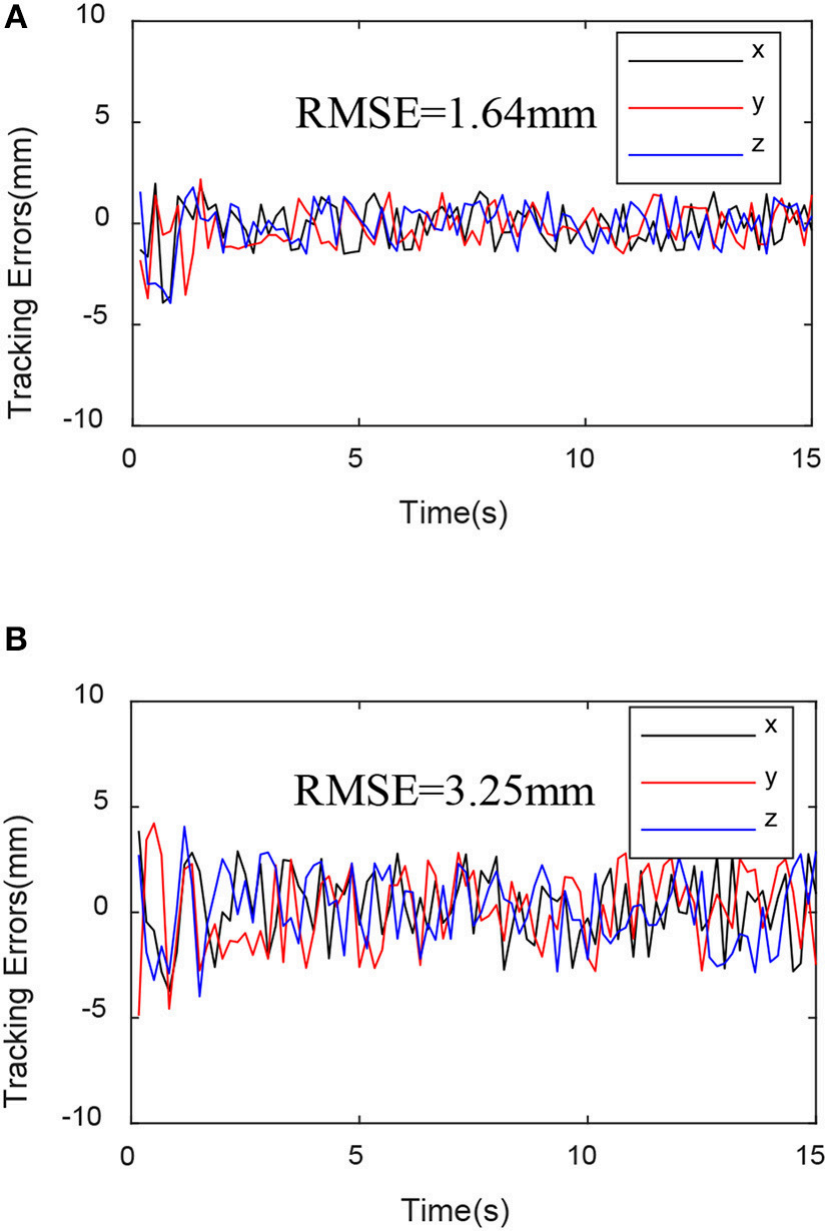
Trajectory tracking variance mean square with disturbance force. **(A)** EKF-based PD controller trajectory tracking variance mean square. **(B)** Sensor-based PD controller trajectory tracking variance mean square.

**Figure 12:**
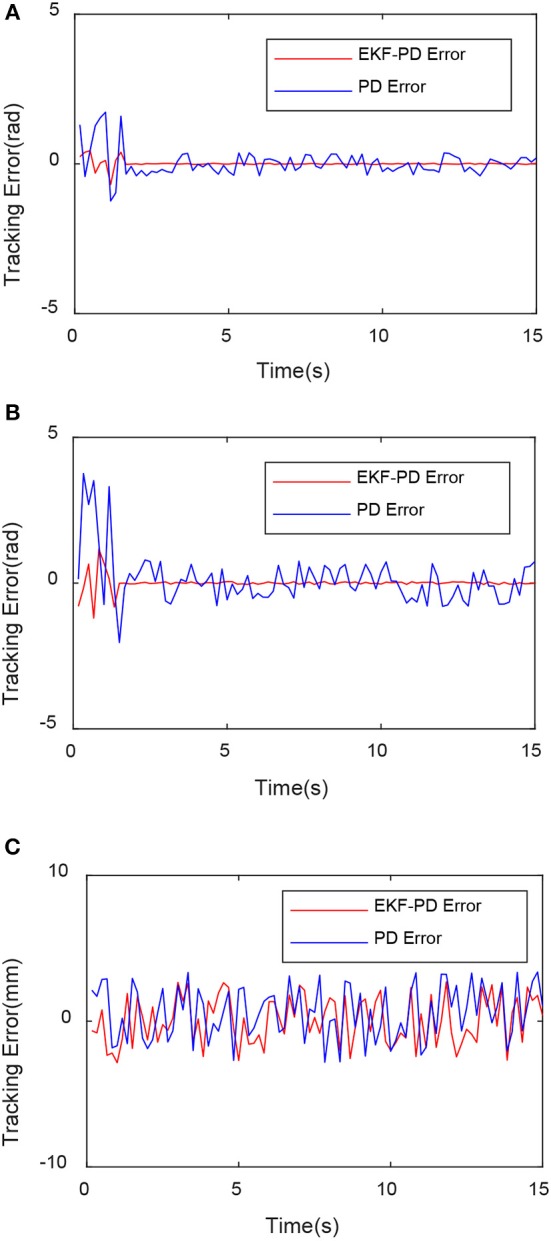
Comparison of tracking error with disturbance force of each joint. **(A)** First joint tracking error comparison. **(B)** Second joint tracking error comparison. **(C)** Third joint tracking error comparison.

The EKF observer in the control system can handle external disturbances in real time. In order to prove its ability to handle real-time interference, a force of sinusoidal variation along the direction of the guide rail is applied at the end of the robot. The force changes are shown in Figure 9. The experimental results are shown in Figures 10–12.

From Figures 10–12, we can see that in the case of external disturbance, the control result based on EKF observer is more stable, and the position deviation of the robot end is almost unchanged, which means that the control based on EKF observer can effectively Handle external interference in real time.

## Conclusion

In this paper, the trajectory tracking control problem of flexible joint robot is discussed. Aiming at the problem that sensor data acquisition is susceptible to interference, an EKF-based PD controller is proposed. The EKF state observer is designed for the control target to observe the output position, and only the position and speed feedback amount of the motor rotor is needed. And the stability analysis of the designed control system is given according to the Lyapunov method. Finally, the effectiveness and superiority of the proposed control algorithm are verified by experiments.

## Data Availability

All datasets generated for this study are included in the manuscript and/or the supplementary files.

## Author Contributions

TM: theoretical analysis. ZS: guide control plan. ZX: guide doing experiment. JD: guide writing paper.

### Conflict of Interest Statement

The authors declare that the research was conducted in the absence of any commercial or financial relationships that could be construed as a potential conflict of interest.
